# Cetuximab-conjugated perfluorohexane/gold nanoparticles for low intensity focused ultrasound diagnosis ablation of thyroid cancer treatment

**DOI:** 10.1080/14686996.2020.1855064

**Published:** 2021-02-01

**Authors:** Ying Liu, Yue Ma, Xiaoshan Peng, Lingling Wang, Haixia Li, Wen Cheng, Xiulan Zheng

**Affiliations:** Department of Ultrasound, Harbin Medical University Cancer Hospital, P.R. China

**Keywords:** Gold nanoparticles, ultrasound diagnosis ablation, thyroid cancer, *in vivo* efficacy, 101 Self-assembly / Self-organized materials, 301 Chemical syntheses / processing, 501 Chemical analyses, 503 TEM, STEM, SEM, 605 Databases, data structure, ontology

## Abstract

We report the formulation of nanoassemblies (NAs) comprising C225 conjugates Au-PFH-NAs (C-Au-PFH-NAs) for low-intensity focused ultrasound diagnosis ablation of thyroid cancer. C-Au-PFH-NAs showed excellent stability in water, phosphate-buffered saline (PBS), and 20% rat serum. Transmission electron microscopy (TEM) images also revealed the effective construction of C-Au-PFH-NAs as common spherical assemblies. The incubation of C625 thyroid carcinoma with C-Au-PFH-NAs triggers apoptosis, as confirmed by flow cytometry analysis. The C-Au-PFH-NAs exhibited antitumour efficacy in human thyroid carcinoma xenografts, where histopathological results further confirmed these outcomes. Furthermore, we were able to use low-intensity focused ultrasound diagnosis imaging (LIFUS) to examine the efficiency of C-Au-PFH-NAs in thyroid carcinoma *in vivo*. These findings clearly show that the use of LIFUS agents with high-performance imaging in different therapeutic settings will have extensive potential for future biomedical applications.

## GRAPHICAL ABSTRACT

**Figure:**
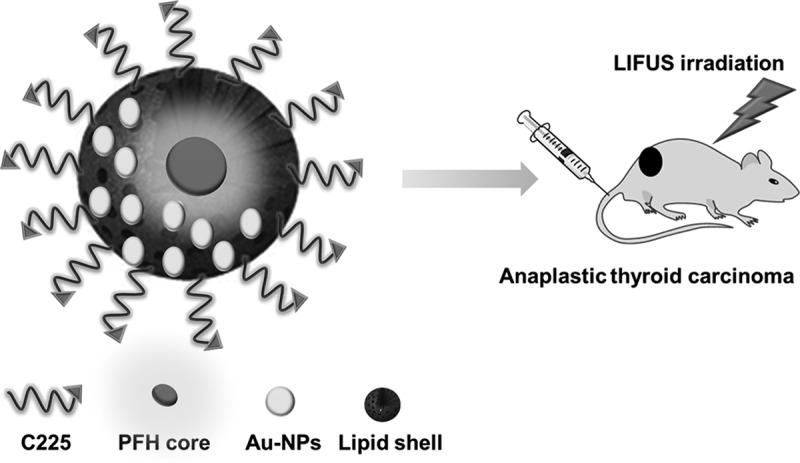

## 1. Introduction

Anaplastic thyroid carcinoma (ATC) is one of the most malignant carcinomas, though it is comparatively rare, characterized by fast proliferation, neck invasion, and remote metastasis [1–4]. The severe prognosis that accompanies ATC is due to the rapid progression of tumours before diagnosis. Current treatment is based on different combinations of chemotherapy and exterior beam radiation, which have been unsuccessful in improving survival, where average survival rates are 4 to 6 months with less than 20% survival in 12 months [5–8]. Therefore, new theranostic approaches for initial detection and efficient ATC treatment are needed [9–11].

Recent studies have extensively explored systems that couple triggerable drug-charged nanocarriers with multiple inner or external stimuli, such as pH, temperature, ultrasound, laser, and microwave radiation, to enable controlled drug release for personalized treatment. These systems have excellent potential for delivering enhanced anticancer treatment, while also decreasing systemic toxicity [12–14]. Low-intensity concentrated ultrasound (LIFUS) has been exhaustively researched for tumour treatment with ultrasound imaging analysis as one of the probable exterior activators, as it is non-invasive and displays significant tissue-penetrating capacity. In particular, it can significantly increase the efficacy of chemotherapy, avoiding harm to nearby cells and reducing adverse side effects [15]. However, the discharge of LIFUS-triggered drugs from nanocarriers for improved tumour therapy is still unsatisfactory, largely due to the comparatively lower accumulation efficiency of nanoparticle-charged nanotransporters at tumour sites. Numerous nanotransporters have been extensively characterized for enhanced aggregation of a large number of tumours while minimizing side effects [15–17].

Several reports have shown that overexpression of the epidermal growth factor receptor (EGFR) is strongly associated with tumour progression, migration, and invasion. EGFR is common in ATC patients [18] and antibodies or small molecules based on EGFR immunotherapy can significantly increase the therapeutic effect against this cancer. A human murine chimeric EGFR-targeted monoclonal antibody called cetuximab has higher specificity for the extracellular domain of human EGFR and inhibits epidermal growth factor signalling in cells by delaying usual receptor function [19–21]. The US Food and Drug Administration approved preclinical treatments using cetuximab for EGFR-expressing cancer tumours, neck, and head carcinomas, and colorectal carcinomas. This C225 antibody might be a suitable objective for modifying the structure of nanocarriers to improve the therapeutic outcome in ATCs. Remarkably, some researchers have revealed that for a wide spectrum of cancers, the blend of C225 with CPT-11 equivalents such as Au-PFH-NAs has significant synergetic antitumour effects [22–25]. Hence, Au-PFH-NAs in combination with C225 could also enhance ATC diagnostics. However, owing to the reduced vascular dispersal of C225 and the hydrophobicity of Au-PFH-NAs, the NAs penetration of the growth and their quantity in the tumour area were inherently imperfect, showing greatly debilitated anticancer efficacy. In contrast, these problems can be minimized by incorporating Au-PFH-NAs and C225 into one nanotransporter to attain a combination chemotherapy while simultaneously providing targeting capability to the nanocarriers [26–29].

Furthermore, medical imaging is essential to early diagnosis and monitoring of tumour progression. Numerous researchers have proposed that LIFUS has the potential to achieve concurrent US medication transfer, meeting the present need for initial treatment and ATC therapy [30–32]. The large and variable dimensions of microbubbles are not appropriate for drug delivery purposes, though they demonstrate outstanding agents for imaging. Realization of the tumour theranostic strategy by conservative US agents may require intensively studied phase-changing NAs that could be activated via LIFUS. Phase-changing NAs provide important benefits in tumour theranostics, facilitating tumour ultrasound and ultrasound-triggered drug release [33–35]. This new strategy offers potential development of malignancy treatments and addresses the present theranostic needs in ATC significantly.

**Figure 1.**
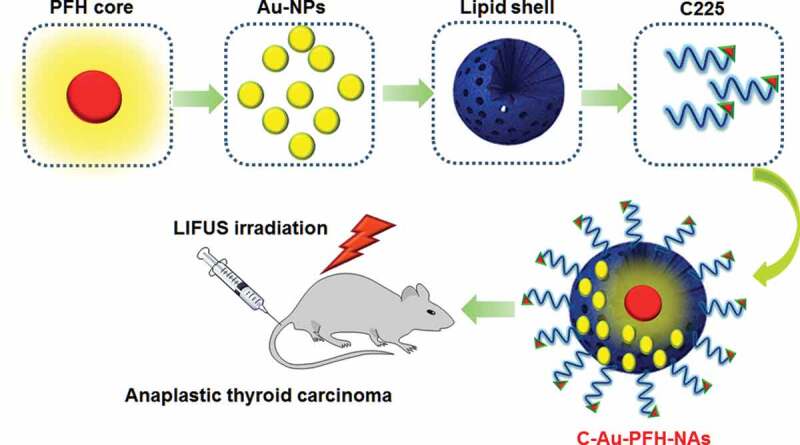
Design and nanoassemblies formulation of C225 conjugates Au-PFH-NAs for safe and efficient *in vivo* drug delivery

The objective of this work was to construct the modification of C225 nanocarrier to exactly prevent ATC that might accrue in cancer cells, in addition to the enhanced permeability and retention (EPR) effect, through the great tumour homing belongings of C225. The Au-PFH-NAs payload could be released and LIFUS-triggered synergistic chemotherapy with C225 may perhaps suggestively make best use of therapeutic efficacy, improve USI and diminish the side effects of chemotherapy. As shown in Figure 1. Due to its tremendous biodegradability and biocompatibility, we used a PHF (perfluorohexane) core as the shell structure of the nanocarrier. We then synthesized phase-changing NAs with perfluorohexane liquid (PHF, 29°C boiling point). Meanwhile, Au-PFH-NAs were burdened into the nanoparticles at the similar period of time as C225 was conjugated on surface of gold nanoparticles afford (C-Au-PFH-NAs) C225-conjugated Au-PFH-NAs-charged phase transformation. To our knowledge, this is the first work of a LIFUS-mediated C225 modified nanosyste that assimilates tumour targeted both US imagery and US activated drug conveyance to ATC.

## 2. Experimental section

### 2.1. Materials

Perfluoropentane (PFP, boiling point of 29°C), *N*-(3-dimethyl-aminopropyl)-*N*′-ethylcarbodiimide hydrochloride (EDC), and fluorescent dyes (1,1′-dioctadecyl-3,3,3′,3′ tetramethylindocarbocyanine perchlorate (DiI), *N*-Hydroxysuccinimide (NHS), Lysotracker and 4′,6-diamidino-2-phenylindole (DAPI)) were purchased from Sigma-Aldrich (St. Louis, MO, USA). Cetuximab (C225, Erbitux) was purchased from Merck KGaA Co. (Frankfurter, Germany). FITC, Tween 80, RPMI-1640 medium (1640) and fetal bovine serum (FBS) was purchased from Abcam Co. (Shanghai, China). Cell Counting Kit-8 (CCK-8) was obtained from Dojindo Molecular Technology (Shanghai, China). Trichloromethane and dimethyl sulfoxide (DMSO) were purchased from Chongqing Chuandong Chemicals (Chongqing, China). All other reagents used in this work were of analytical grade and were used as received.

### 2.2. Synthesis of C-Au-PFH-NAs

Folate-targeted gold (Au) and perfluorohexane (PFH) nanoparticles (Au-PFH-NAs) were fabricated by a film hydration method coupled with a double emulsion method. At first, 100 mg of lipids compounds including dipalmitoylphosphatidylcholine (DPPC), 1,2-distearoyl-sn-glycero-3-phosphoethanolamine-N-[folate(polyethylene glycol)-2000] (DSPE–PEG (2000)-folate), 1,2-Dipalmitoyl-sn-glycero-3-phosphoglycerol (DPPG), and cholesterol (CH) with mass ratios of 5: 2: 1.5: 1.5 were dissolved in 10 mL aqueous solution until clear. Then, 100 mL of Au (10 mg/mL) was added to the aqueous solution. The mixture was transferred into the rotary evaporator (Yarong Inc., Shanghai, China) and rotated at 55 rpm and 45°C to clear the aqueous solution. The thin lipid film formed was rehydrated with 5 mL PBS to generate a brown suspension. The suspension was then dispersed with a high-speed homogenizer (FJ300-SH, Shanghai, China) for 5 m after drop-by-drop addition of 500 µL PFH. The secondary emulsion was performed by means of an ultrasonic oscillator (SONICS & MATERIALS Inc., USA) for 5 m in an icecold environment (0°C). Finally, the mixture of nanoemulsions was harvested and centrifuged (Eppendorf, Germany) at 4500 rpm for 5 m and washed with deionized water for three times to sweep away dissociative Au and PFH. The final emulsion was collected and stored at 4°C for further use. Fluorescent nanoemulsions were obtained according to the above procedure except that the DiI was blended in the lipids solution.

### 2.3. C225 conjugation

Conjugation of C225 to the Au-PFH-NAs loaded nanoparticles was performed using carbodiimide chemistry. Briefly, the prepared Au-PFH-NAs were dissolved in 5 mL of MES buffer solution (0.1 M, pH 5.5) together with a mixture of 3 mg of EDC and 10 mg of NHS, and then incubated vigorously for a period of 1 h on a gentle shaker. The resulting solution was centrifuged and washed three times with PBS to remove unreacted 1-ethyl-3-(3-dimethylaminopropyl)carbodiimide (EDC) and 10 mg of N-hydroxysuccinimide (NHS). Then, the sediment was redissolved in 5 mL of 2-(N-morpholino)ethanesulfonic acid (MES) buffer solution (0.1 M, pH 8.0). Next, excess C225 was dropped into the above solution and stirred on a gentle shaker for another 2 h. After the reaction was completed, Au-PFH-NAs with C225 conjugation (C-Au-PFH-NAs) were obtained by centrifugation, washed thrice with PBS again to remove unconjugated C225 and preserved at 4°C before use. All the aforementioned procedures were carried out in an ice bath.

### 2.4. Characterization of C-Au-PFH-NAs

Optical microscopy (CKX41; Olympus, Tokyo, Japan) and confocal laser scanning microscopy (CLSM) (Nikon A1, Tokyo, Japan) were employed to observe the morphology and particle distribution of Au-PFH-NAs and C-Au-PFH-NAs. A dynamic light scattering analyzer (DLS) (Malvern Instruments, Malvern, UK) was used to determine the mean particle size and polydispersity index (PDI) NAs. Morphological characterization of NAs was carried out by transmission electron microscopy (TEM; H-7500; Hitachi, Tokyo, Japan). The mean particle size of the nanoparticles was determined by DLS within 7 days to evaluate the stability of the Au-PFH-NAs and C-Au-PFH-NAs.

### 2.5. Cell culture and nude mice

The Cell Bank of the Chinese Academy of Sciences (Shanghai, China) acquired a human anaplastic thyroid carcinoma line (C643). The cells were grown in medium RPMI-1640 containing 10% FBS and 1% penicillin-streptomycin at 37°C in humidified air with 5% CO_2_. At the Laboratory Animal Center of Department of Ultrasound, Harbin Medical University Cancer Hospital (Harbin, China), BALB/C Female both mice and nude mice (balancing about 19 g, 25 days) were bought then raised. All animals on our studies were collected from the Harbin Medical University Cancer Hospital Laboratory Animal Center and retained in accordance with rules authorized by the Harbin Medical University’s Animal Ethics Committee (Harbin, China). Furthermore, all animal experimental activities were strictly in line with the policy of the Harbin Medical University’s Institutional Animal Care and Use Committee (IACUC), and this study was endorsed by the IACUC.

In order to start an ATC model in nude mice, C643 cells were collected, splashed thrice with the FBS-free medium of RPMI-1640, and subcutaneously inoculated into each mouse’s left flank (3 **×** 10^7^ C643 cells in 150 μL FBS-free medium of RPMI-1640 for each mouse. A Vernier caliper was used to measure the length and width of the tumour and the tumour quantity was considered by the calculation: volume-(length as width**×** 2)/2.

### 2.6. *In vitro* analysis

#### 2.6.1. *In vitro* intracellular uptake C-Au-PFH-NAs

In cultivation dishes, seeded the C643 cells for CLSM at a mass of 1 × 10^6^ cell mL/dish, grown at 37°C in moistened air comprising 5% CO_2_. The cells were spilt into four groups after 24 h of culture: C-Au-PFH-NAs were handled, respectively, with 10 min and 15 min Dil- labeled C-Au-PFH-NAs (1 mg/mL), and after blocking the cells were washed three times with phosphate-buffered saline (PBS). Then, Dil- labeled C-Au-PFH-NAs (1 mg/mL) incubated the cells. The cells were washed with PBS three times after 2 h incubation with nanoparticles, fixed with 4% paraformaldehyde (200 μL) for 15 minutes, and then gestated by 6-diamidino-2-phenylindole (DAPI) (10 μg/mL, 200** **μL) for 20 min. Lastly, CLSM pictured the dishes [36–38].

#### 2.6.2. *In vitro* cytotoxicity assay

The CCK-8 assay assessed the cell viability. C643 cells were seeded into 96-well plates (1 × 10^3^ cells per well, 100 μL). After 24 h’ incubation to assess the cell viability Au-PFH-NAs and C-Au-PFH-NAs treated at levels of 10, 5, 2.5, 1.25, 0.625, and 0.312 µM for 24 hours. Au-PFH-NAs and C-Au-PFH-NAs cells were incubated for 24 hours. The positive control used as the untreated C643 cells. The *in vitro* cytotoxicity assay performed and the calculated made by the company manufactures guidelines.

#### 2.6.3. Apoptosis examinations

The cells were seeded (4 × 10^6^ C643 cells per well, 1.5 mL) into a 6-well dish and grown at 37°C in a humidified incubator with 5% CO_2_ for 24 hours. The IC_50_ concentration used by Au-PFH-NAs and C-Au-PFH-NAs. The cell apoptosis assay grouping technique was in accordance with the cell viability assay group. After administering IC_50_ concentration of the formulations of Au-PFH-NAs and C-Au-PFH-NAs was implemented 2 hours later [39–41].

#### 2.6.4. Cell cycle arrest examinations

The cells were seeded (4 × 10^6^ C643 cells per well, 1.5 mL) into a 6-well dish and grown at 37°C in a humidified incubator with 5% CO_2_ for 24 hours. The IC_50_ concentration used by Au-PFH-NAs and C-Au-PFH-NAs. The cells were gathered and analyzed in the PI-stained cells after 24 hours of culture, and the percentages of the cells in the G0/G1, S phase, and G2/M phases were evaluated [42–44].

#### 2.6.5. *In vitro* fluorescence imaging in xenografts tumour

A continuous dosage of DiR labelled Au-PFH-NAs and C-Au-PFH-NAs (2 mg/mL, 200 μL) was given to C643 tumour-bearing mice. With 1% pentobarbital, all mice were totally narcotized and fluorescence pictures were acquired before injection and 3 h, 6 h and 24 h post-injection. A vivid fluorescence imaging for tiny animals evaluated the fluorescence intensity changes in the tumour areas *in vivo*. For *ex vivo* fluorescence imaging, the significant organs and tumour of one mouse were gathered. In addition, Dil-labeled Au-PFH-NAs and C-Au-PFH-NAs (2.5 mg per mL, 150 μL) were injected through the intravenous of C643 tumour-bearing mice were injected 6 hours after injection. At the predetermined post-injection moment, tumour matters and significant tissues were gathered, segmented, and ice-covered. DAPI dyeing was conducted in the dark for 5 min after fastening with 4% paraformaldehyde. The biodistribution of Dil-labeled Au-PFH-NAs and C-Au-PFH-NAs was monitored by CLSM [45–47].

### 2.7. Therapeutic efficacy *in vivo*

When the subcutaneous tumour reaches 100 mm^3^ in volume, an antitumour assay was conducted on xenografts of mice carrying anaplastic thyroid cancer. The tumour-bearing mice were arbitrarily split into three communities (n-5 per unit): control group (saline) and free Au-PFH-NAs and C-Au-PFH-NAs were administered. 200 µL of the blend was injected with the same dose of Au-PFH-NAs and C-Au-PFH-NAs (1 mg/kg) through the tail vein in a 1% saline solution were determined 6 hours after injection with the US agent filling the investigation with the tumour superficial. Afterward the inoculations of C643 cells, 5 consecutive treatments were performed each 72 hours starting on day 20 and ending on day 37. Each mouse’s tumour dimensions and weight was recovered every three days, and changes in tumour volume were examined from the relative tumour dimensions V/V_0_ (V_0_: initial volume prior to treatment), and tumour growth curves were drawn at the same time. On day 21 days, all mice were euthanized and dissected and weighed the tumour masses. In addition, studies in histology and immunohistochemistry were conducted. Sections of the tissue were stained with histopathology [48–50].

### 2.8. Statistical assay

Each experiment was repeated at least three times. The mean ± standard deviation of all the data was analyzed in the tables and figures. One-way ANOVA was utilized for the inter-group comparison, and the bilateral paired *t*-test and the least significant difference (LSD) test were utilized for statistical evaluation. *P* values <0.05 were considered statistically significant (*p < 0.05, **p < 0.01, ***p < 0.001, ****p < 0.0001).

## 3. Results and discussion

### 3.1. C-Au-PFH-NAs characterization

**Figure 2.**
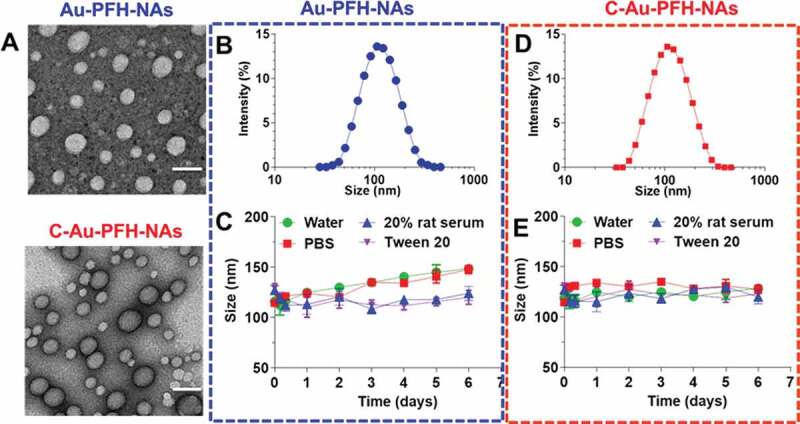
(a) TEM image of Au-PFH-NAs and C-Au-PFH-NAs. Scale bars, 100 nm (b and d) DLS image of Au-PFH-NAs and C-Au-PFH-NAs, respectively. (c and e) Stability of Au-PFH-NAs and C-Au-PFH-NAs, respectively, in water, PBS, 20% rat serum, and Tween-20 at 37°C

With Au-PFH-NAs and C-Au-PFH-NAs in hand, we examined these compounds using TEM analysis (Figure 2). We next tested whether they were able to recapitulate the expected self-assembly behaviour in aqueous solutions. For this purpose, we dissolved the C-Au-PFH-NA prodrugs in dimethyl sulfoxide (DMSO) (10 mg/mL) and then rapidly injected them into deionized (DI) water under ultrasonication. This procedure allowed us to verify that the solution was transparent and slightly bluish. Electron microscopy revealed that the drug molecules self-assembled to form a spherical nanoparticle structure and DLS showed a single peak distribution of the nanoparticles. The average hydrodynamic diameter (intensity) of compound 1 was approximately 107.1 nm, while the diameter of compound 2 was approximately 108.0 nm (Figure 2(b)). However, adhesion is observed between nanoparticles formed upon self-assembly with simple small-molecule drugs [51–53]. Therefore, we generate miscible gold nanoparticles by combining the prodrug with the appropriate amount of C225 molecules. These nanoassemblies are formed and have been widely used for *in vivo* drug delivery in order to solve the problem of adhesion and optimize cancer-specific drug delivery. Next, we measured the stability of C-Au-PFH-NAs in various solvents, such as water, PBS, 20% rat serum, and tween-20, and found a significantly stable size in each (Figure 2(c)). Taken together, these results suggest that although C-Au-PFH-NAs can self-assemble to form nanoparticles, they may not be sufficiently stable. Therefore, C225 nanoparticles loaded with Au-PFH were investigated further to evaluate their anticancer efficacy *in vitro*.

### 3.2. *In vitro* cell experiments

#### 3.2.1. *In vitro* intracellular uptake

**Figure 3.**
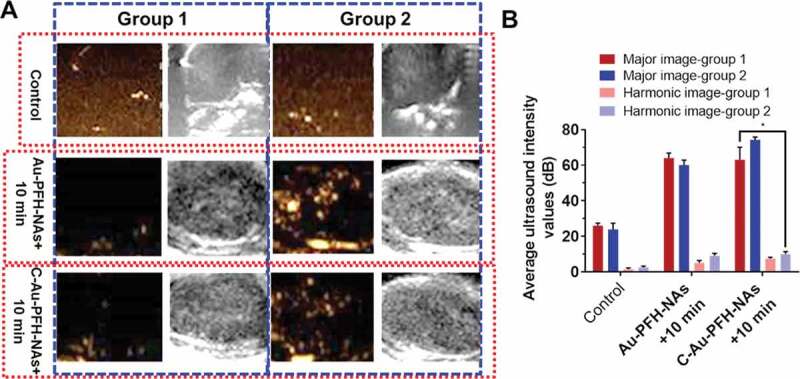
Cellular uptake of control NAs and C-Au-PFH-NAs with 10 and 15 minute intervals. Scale bar 20 μm

As illustrated in Figure 3 and Figure S1, a much stronger red fluorescence was derived from cells transfected with Dil-labeled C-Au-PFH-NAs for 10 and 15 minutes [54]. Furthermore, a stronger red fluorescence was noted in cells after exposure to the C-Au-PFH-NAs group. These findings revealed that the elevated tumour-homing characteristics of C225 allowed the C-Au-PFH-NAs to fix tightly to the C643 cells, facilitating intracellular uptake considerably. In the resentment group, C-Au-PFH-NAs lost the capacity to target the C643 cells because surplus-free C225 led to congestion, resulting in low levels of C-Au-PFH-NAs around the cells and demonstrating that the EGFR-mediated targeting was effective in C-Au-PFH-NAs.

#### 3.2.2. *In vitro* cytotoxicity assay

**Figure 4.**
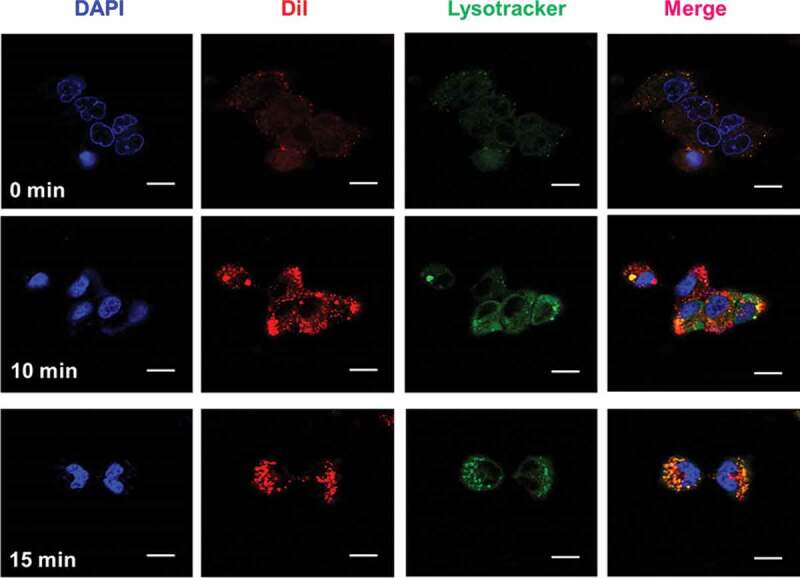
(a) *In vitro* cytotoxicity against C643 thyroid carcinoma cell. (b) Flow cytometry analysis of Au-PFH-NAs and C-Au-PFH-NAs. (c) Quantification of apoptosis by flow cytometry analysis. (d) Cell cycle arrest of Au-PFH-NAs and C-Au-PFH-NAs. (e) quantification of cell cycle arrest. *P* values < 0.05 were considered statistically significant (**p < 0.01, ***p < 0.001)

The CCK-8 assay was used to assess cell viability with different NP formulations at multiple concentrations, showing a dose-dependent model. As illustrated in Figure 4(a), the cell viability of nanoparticles in the analyzed dose range was more than 80%, with concentrations ranging from 0.312 to 0.625 μM. The comparatively small (insignificant) loss of viability suggested that the elevated biocompatibility of these phase-changing nanoparticles was appropriate for *in vivo* applications. As expected, Au-PFH-NAs and C-Au-PFH-NAs cell viabilities decreased considerably as the levels of C-Au-PFH-NAs increased. In particular, the viability of cells treated with C-Au-PFH-NAs was lower than Au-PFH-NAs at the same concentration, implying that the mixture in C-Au-PFH-NAs could boost cytotoxicity synergistically. The remarkably improved cytotoxicity of C-Au-PFH-NAs may be due to the increased cell membrane permeability, caused by the cavitation effect, and the improved C-Au-PFH-NAs at the target site, which significantly increased the inhibitory effect of C-Au-PFH-NAs on cell development.

#### 3.2.3. Cell apoptosis and cell cycle assay

Next, we evaluated cellular apoptosis. In the groups assessed, increasing amounts of total apoptosis (TA) were observed when comparing the control cells, cells treated with Au-PFH-NAs, and cells treated with C-Au-PFH-NAs, respectively (Figure 4(b)). It should be noted that the proportion of apoptotic cells in the Au-PFH-NAs treated group was lower than that of those in the C-Au-PFH-NAs group, whereas it was significantly greater than that of the control cells. Cell cycle assays were also carried out to evaluate whether the anti-proliferation observed with Au-PFH-NAs and C-Au-PFH-NAs treated cells involved changes in the cell cycle. A higher percentage of the G2/M phase cells was observed in all treatment groups compared to the control group (Figure 4(d,e)). Cells treated with Au-PFH-NAs were arrested in the G2/M phase more often than those in the control group, but less frequently than those in the C-Au-PFH-NAs, which is consistent with the results of cytotoxicity and apoptosis assays mentioned above. Hence, we detected an increased proportion of cell cycle arrest in the G2/M phase of treated cells, and the distinct arrest stages can be ascribed to the specific effects among distinct tumour cells. Hence, C-Au-PFH-NAs showed important cell cycle arrest, impacting the growth of C643 cells during the G2/M phase.

Complete analysis of the experimental outcomes *in vitro* clearly indicated that nanoparticles could act as exceptional vehicles for Au-PFH-NAs and C-Au-PFH-NAs, and the combination of C225 enhanced the targeted cell recognition and endocytosis, improving the therapeutic effect of C-Au-PFH-NAs by maximizing inhibition of cell proliferation. This may be due to increased cell membrane permeability, caused by cavitation and UTMD, and the improved release of C-Au-PFH-NAs at the target site, which significantly increased inhibition of tumour cell proliferation.

### 3.3. *In vivo* fluorescence imaging in xenografts tumour

**Figure 5.**
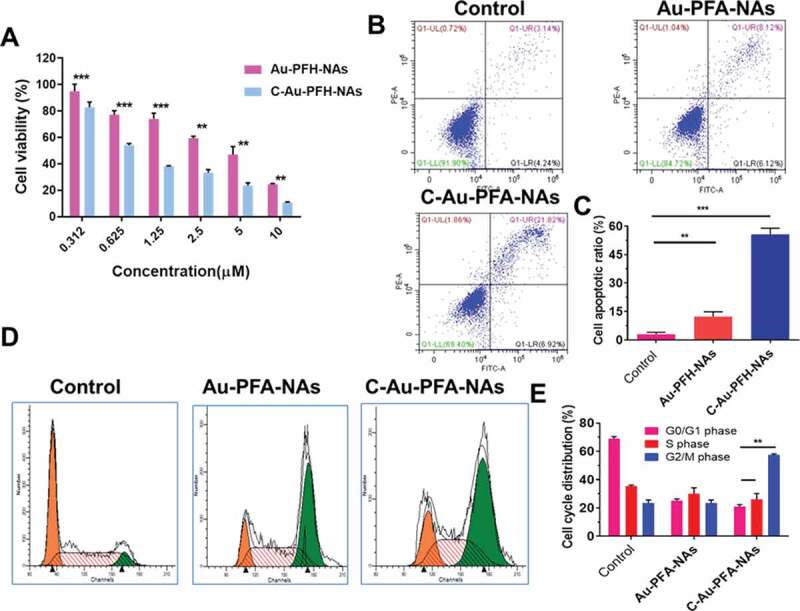
*In vivo* pharmacokinetics and biodistribution studies of Control, Au-PFH-NAs and C-Au-PFH-NAs. (a) *In vivo* plasma of the drugs following intravenous injection of Control, Au-PFH-NAs and C-Au-PFH-NAs. (b) The biodistribution studies Au-PFH-NAs and C-Au-PFH-NAs. (c) The intensity of the DiR label Au-PFH-NAs and C-Au-PFH-NAs. *P* values < 0.05 were considered statistically significant (*p < 0.05)

In order to assess the targeting effectiveness and biodistribution of Au-PFH-NAs and C-Au-PFH-NAs *in vitro*, fluorescence imaging was implemented at prearranged period opinions. Compared to the non-targeted group’s small fluorescence signal at each stage in time, the targeted group’s important accumulating fluorescence signal seemed at the tumour place and peaky at 6 hours at values 6 fold greater than control group (4 ± 1) sometimes 10^9^ vs. (9 ± 1), respectively, 108 (ps^−1^ cm^−2^ sr^−1^)/(µW cm^−2^) (Figure 5(a)). The intensity of the fluorescence removed tumour and significant tissues were examined after 24 h ex vivo, and the tumour intensity fluorescence in the selected group was still 2 fold greater than that in the non-target group (3.0 ± 0.4) sometimes 10^8^ vs. (2.1 ± 0.3), respectively, 10^8^ (ps^−1^ cm^−2^ sr^−1^)/(µW cm^−2^). There was virtually no distinction between the targeted and non-targeted groups in the fluorescence intensified in the significant bodies. Meanwhile, considerably greater fluorescence red signals were noted in the targeted group’s tumour cryosections at 6 hours under CLSM following tumour tissue ultrathin segmenting relative to the less signals red in the non-target group. The aforementioned should be noted that in the tumour cryosections of the Au-PFH-NAs group the red fluorescence signals were significantly improved compared to that of the C-Au-PFH-NAs group after irradiation. The distribution of the fluorescence signal in the main organs showed no important alteration in both group but primarily in the liver and spleen (Figure 5(b,c)). The non-targeted group’s low fluorescence signals at the tumour place may outcome from the EPR effect, which facilitated inert combination in tumour tissues, whereas the targeted group’s greater fluorescence signal was primarily due to the C225-mediated endocytosis mechanism. In addition, C-Au-PFH-NAs could overcome tumour biological barriers, and the accumulation of C-Au-PFH-NAs at the objective places was endorsed after microbubble oscillation, cavitation and destruction. During the process of the oscillation and crash of the acoustic microbubble by the US targeted microbubble removal impact, the cell membrane could be interrupted and the permeability enhanced, allowing more effective accumulation of C-Au-PFH-NAs at the objective locations. These conclusions more confirmed that C225 might precisely effort nanocarriers to tumour cells, preventing them from rapidly re-entry into systemic circulation and this allowing extravascular diagnosis and efficient antitumour therapy with an agent.

### 3.4. *In vitro* ultrasound imaging

**Figure 6.**
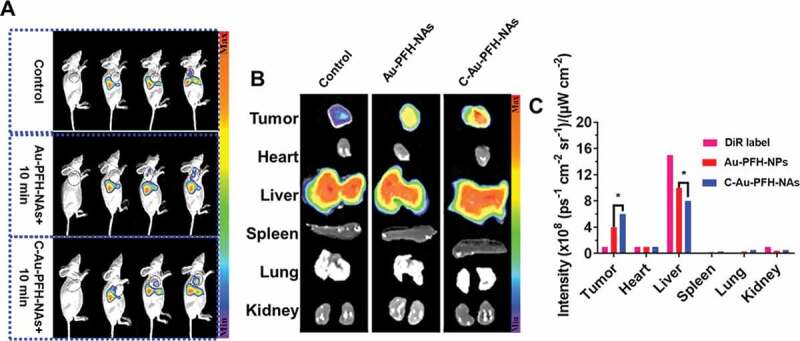
(a) Ultrasound image. (b) The average intensity values of Au-PFH-NAs and C-Au-PFH-NAs. *P* values < 0.05 were considered statistically significant (*p < 0.05)

Based on Au-PFH-NAs and C-Au-PFH-NAs targeted accumulation capacity in tumour cells, we gambled that the phase changing nanoparticles can aid as US contrast to improve USI and treatment scratches [55–57]. Following the administration of various medicines before LIFUS irradiation, even less or anechoic and less contrast improved US signals were noted in each group (Figure 6(a)). Six hours after the administration of various treatments, LIFUS was performed in all groups same time periods with *in vivo* ultrasound imaging. In comparison with the saline, expressively sturdier spot like echo signs slowly accrued in both modes at the tumour places in the treated group, while no evident deviations were detected in the saline group, and only negligible signs looked in the non-target group. This outcome recommended that C225 eased the directing of tumour tissue accretion, and huge quantities of microbubbles were produced when phase-changing NAs were subjected to ADV at the LIFUS triggered tumour site, resultant in improved US imaging. Though owing to the absence of C225-mediated targeting capacity, the Au-PFH-NAs and C-Au-PFH-NAs inadequate ADV could not effectively improve ultrasound imaging. Furthermore, apparent enrichment without LIFUS irradiation was not found in the Au-PFH-NAs and C-Au-PFH-NAs alone could not *in vitro* improve the ultrasound imaging shown in Figure 6(b). These findings showed that because of their relative stability, Au-PFH-NAs and C-Au-PFH-NAs were appropriate as ultrasound imaging agents and efficient *in vivo* nanocarriers. The above information were compatible with the outcomes of ultrasonic imaging, additional checking the effectiveness of the beleaguered ultrasonic of C-Au-PFH-NAs lower than LIFUS irradiation and local LIFUS radioactivity can boost the precision of phase-changing C-Au-PFH-NAs.

### 3.5. Therapeutic efficacy *in vivo*

**Figure 7.**
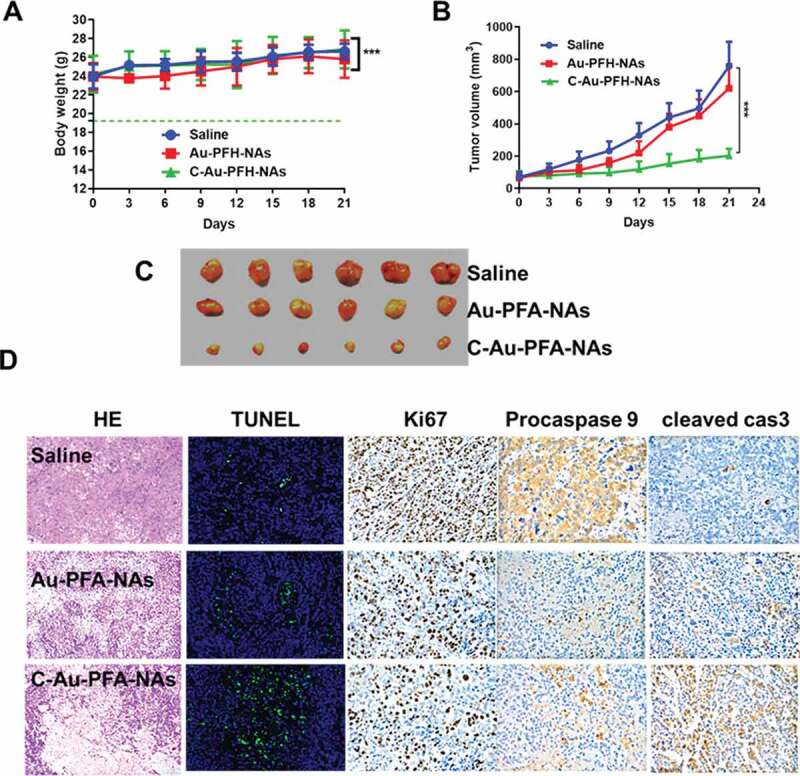
(a) Body weight (g), (b) Tumour volume (mm^3^). (c) Morphology of the tumours 21 days after treatment. The tumour volume, tumour weight, and average body weight. (d) Representative HE, TUNEL, Ki67, procaspase 9, and cleaved caspase 3 analysis of the tumours excised from the treated groups after injection of the drugs. The image magnification is x400. *P* values < 0.05 were considered statistically significant ***p < 0.001)

To establish the potential of Au-PFH-NAs and C-Au-PFH-NAs to be translated to clinical applications, we assessed the antitumour efficacy *in vivo* was explored in subcutaneous C643 models, interested by the notable therapeutic efficacy of the mixture of Au-PFH-NAs and C-Au-PFH-NAs *in vitro*. In order to demonstrate the therapy impact, numerical data for separate groups of mice were drawn (Figure 7(a-c)). The therapeutic effectiveness was evaluated by tracking changes in each group’s tumour volume. The tumour in the saline groups the debauched and there was no significant decrease in the tumour dimensions in the C-Au-PFH-NAs group, indicating that the dose of C-Au-PFH-NAs was dependable *in vivo* and the well-known epidermal growth factor is target for tumour cell identification and treatment. But, C-Au-PFH-NAs accumulation at the tumour site depended solely on the existence of vessel fenestrations and vascular leakage, and inadequate drug release from the tumour site restricted the therapeutic effect. These findings showed that C-Au-PFH-NAs in nude mice could additional enhance the therapeutic effect of anaplastic subcutaneous thyroid cancer. Simultaneously, compared with control (saline) groups, H&E, procaspase 9 (brown), cleaved-caspase 3 (brown) expression levels are enhanced. Then, the Ki67 staining and TUNEL assay were showed to measure the apoptosis of the tumour *in vivo* (Figure 7(d)) Furthermore, during the course of therapy, C-Au-PFH-NAs show no statistically important distinction in body weight between all mice groups. But, Au-PFH-NAs significantly reduce body weight. The results of procaspase 9, cleaved caspase 3 histopathology analyses were consistent with the results of these therapeutic studies, showing extensive apoptosis and reduced cell proliferation caused by the C-Au-PFH-NAs treatments (Figure 7(d)). The above findings obviously showed that in nude mice, the combination of C-Au-PFH-NAs attained a notable excellent therapeutic effect counter to ATC, importance the security of beleaguered tumour treatment. This diagnostic approach is a preferred method for ATC of the thyroid, significantly improving the healing capacity lacking noticeable side effects.

## 4. Conclusion

The data presented here highlight a strategy and rationale for improving both the safety and effectiveness of Au-PFA-NAs. As the synthetic Au-PFA-NAs and C-Au-PFA-NAs are fully biocompatible composites with minimal modifications, the safety risks have been minimized when considering their clinical translation. Furthermore, given the ability of Au-PFA-NAs to overcome the cetuximab (C225)-conjugated C-Au-PFA-NAs, our approach is expected to have high value as an optional therapeutic platform for treating patients with drug-resistant cancer. Lastly, in addition to taxane agents, we envision that this C-Au-PFA-NAs-based approach could be a simple, yet broadly applicable strategy for improving cytotoxic nanotherapeutics with other antitumour agents.

## Supplementary Material

Supplemental Material

## Data Availability

All data and material are included in the article and its additional files.
