# Supplementary material for: Cetuximab-conjugated perfluorohexane/gold nanoparticles for low intensity focused ultrasound diagnosis ablation of thyroid cancer treatment
Source: Sci Technol Adv Mater. 2021 Feb 1;21(1):856–66. doi: 10.1080/14686996.2020.1855064 (PMC7850351; doi:10.1080/14686996.2020.1855064)
Supplement: Supplemental Material [file TSTA_A_1855064_SM1077.pdf]

Cetuximab-Conjugated Perfluorohexane/Gold Nanoparticles for Low Intensity Focused Ultrasound Diagnosis Ablation of Thyroid Cancer Treatment

Yue Ma, Xiaoshan Peng, Lingling Wang, Haixia Li, Wen Cheng, Xiulan Zheng, Ying Liu*

Department of Ultrasound, Harbin Medical University Cancer Hospital, PR. China.

Correspondence

Prof. Dr. Ying Liu

No.150, Haping Road, Harbin 150081, P.R. China

E-mail - liuying865@yahoo.com

Telephone–037123906685

Fax- 037123906685

Information Classification: General

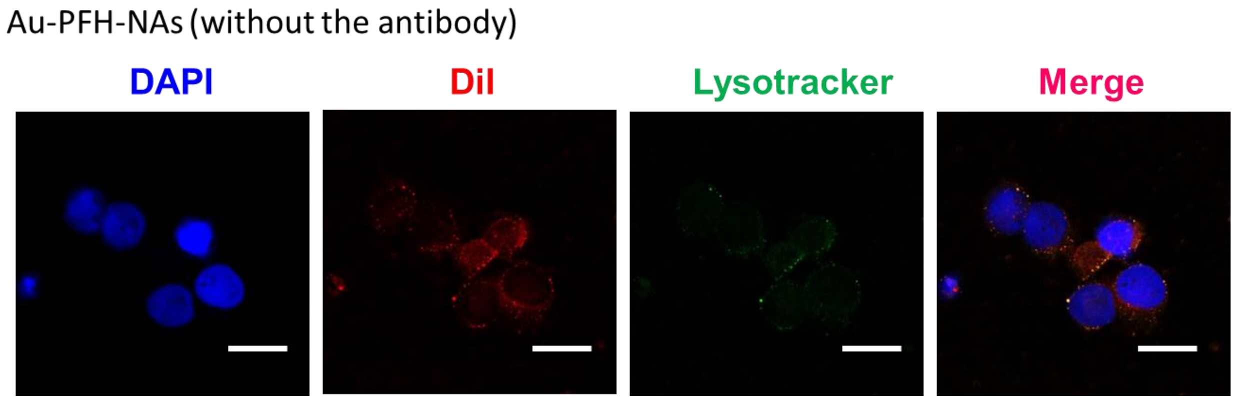

Figure S1. Cellular uptake of Au-PFH-NAs without antibody. Scale bar 20 μm.

Information Classification: General
